# Comment on “Impact of Diabetic Foot on Selected Psychological or Social Characteristics”

**DOI:** 10.1155/2014/246403

**Published:** 2014-10-28

**Authors:** Vladimíra Fejfarová, Alexandra Jirkovská, Eva Dragomirecká, Frances Game, Robert Bém, Michal Dubský, Veronika Wosková, Marta Křížová, Jelena Skibová, Stephanie Wu

**Affiliations:** ^1^Diabetes Center, Institute for Clinical and Experimental Medicine, Videnska 1958, 140 21 Prague 4, Czech Republic; ^2^Department of Social Work, Faculty of Arts, Charles University, 116 42 Prague, Czech Republic; ^3^Diabetes Unit, Derby Hospitals NHS Foundation Trust, Derby DU22 3NE, UK; ^4^Center for Lower Extremity Ambulatory Research, Dr. William M. Scholl College of Podiatric Medicine, Rosalind Franklin University of Medicine and Science, Chicago, IL 60064, USA

The interesting comments of Assistant Professor Cakir et al. [1] highlight the rather surprising results of our study, which show a relatively low incidence of severe forms of depression or any associated consequences. A query of the authors was directed at the possible effect of antidepressive treatment on the results of our work.

Our study showed a relatively high incidence of mild forms of depression in both groups of patients (patients with diabetic foot versus diabetic controls [2]). Severe forms of depression were not as frequent as we originally supposed. A similar occurrence of forms of depression was detected in a special subgroup consisting of patients with previous major amputations. During the study, the pharmacological medication and a history of used drugs potentially affecting the psychological state of individuals were investigated in all study subjects using the questionnaire method. Only very small groups of patients, specifically 7.7% of patients with diabetic foot and 6.25% of diabetic controls, were treated with antidepressants. The difference in use was not statistically significant.

Based on our data, we will support further investigation of psychosocial assessments in patients with the diabetic foot to improve their standard of living and therefore their quality of life.
